# Disparities in Access to Group Parenting Training Programmes: A Cross-Sectional Analysis of Local Authorities Across England

**DOI:** 10.1192/bjo.2024.153

**Published:** 2024-08-01

**Authors:** Rosemary Gorringe, Peter Woods, Manuel Giardino, Juan Luque Solano, Nathan Hodson

**Affiliations:** 1 Warwick Medical School, Coventry, United Kingdom; 2 University of Buckingham Medical School, Buckingham, United Kingdom

## Abstract

**Aims:**

To compare the funding, courses and delivery modalities of parenting training delivered across London borough councils, metropolitan district councils, and county councils in England.

**Methods:**

Freedom of Information requests were piloted on 5 local authorities. Following optimisation, requests were sent out to 74 local authorities across England requesting information on funding for parenting training programmes (26 London Borough Councils, 16 County Councils, and 29 Metropolitan Borough Councils). 26/32 London Boroughs, 16/21 County Councils, and 29/36 Metropolitan Boroughs were sent requests. No follow-up emails were sent chasing responses; however, clarification was provided where necessary. Data were analysed on Excel to observe patterns and disparities.

**Results:**

We received responses from 74 local authorities, and 50 were usable. The mean amount of funding spent across local authorities was £881,254 (standard deviation 1,627,921). There were 18 parenting programmes used, the most common was Triple-P. The average number of parents supported by parenting programmes per local authority was 949 (standard deviation 1410). Local authorities reported spending an average of £27,430 (standard deviation 41005) on digital parenting programmes. The mean number of parenting staff was 36 (standard deviation 59).

**Conclusion:**

We found wide disparities in spending, staffing, and programme choices representing a fragmented landscape of parenting training provision. Several local authorities could not separate spending on parenting training, and parental engagement was not reported consistently. We recommend more consistent reporting of parental initiation, engagement, and completion of training programmes to ensure equitable access and provision of parenting training nationwide.

